# A case report of secondary synchronous diagnosis of multiple myeloma and systemic lupus erythematosus after breast cancer treatment: A CARE-compliant article

**DOI:** 10.1097/MD.0000000000030320

**Published:** 2022-09-02

**Authors:** Pei-Hsin Chen, Heng-Hsin Tung, Chin-Husan Lin, Kuan-Po Huang, Yung-Lun Ni, Chin-Yao Lin

**Affiliations:** a Department of Nursing, Taichung Tzu Chi Hospital, Buddhist Tzu Chi Medical Foundation, Taichung City, Taiwan; b School of Nursing, National Yang Ming Cho Tung University, Taipei City, Taiwan; c Department of pathology, Taichung Tzu Chi Hospital, Buddhist Tzu Chi Medical Foundation, Taichung City, Taiwan; d Department of hematology, Taichung Tzu Chi Hospital, Buddhist Tzu Chi Medical Foundation, Taichung City, Taiwan; e Department of chest medicine, Taichung Tzu Chi Hospital, Buddhist Tzu Chi Medical Foundation, Taichung City, Taiwan; f Department of Breast Medical Center, Taichung Tzi-Chi Hospital, Buddhist Tzu Chi Medical Foundation, Taichung City, Taiwan.

**Keywords:** breast cancer, case report, lupus erythematous, multiple myeloma, synchronous diagnosis

## Abstract

Breast cancer (BC) is the most diagnosed cancer worldwide. Multiple myeloma (MM) is a hematologic malignancy characterized by the overproduction of monoclonal antibodies in the bone marrow. Systemic lupus erythematosus (SLE) is distinguished by the aberrant activity of the immune system with heterogeneous clinical manifestations. The coexistence of more than one major illness in a patient can present a diagnostic challenge for clinical physicians, especially when the comorbid diseases share a similar clinical presentation. Herein, we report an unusual case of secondary synchronous diagnosis of MM and SLE after BC treatment.

**Patient concerns:**

A 69-year-old female patient with breast cancer experienced severe skin itching and rashes on the face, anterior chest wall, back, and trunk for two days before admission. She had high levels of immunoglobulin and anti-nuclear antibodies; low levels of complements 3 and 4; positive anti-cardiolipin-IgM, anti-beta 2 glycoprotein-1 (anti-β2GP1) antibodies, and lupus anticoagulant results at serological testing.

**Diagnosis:**

The postoperative pathology report showed ductal carcinoma in situ in the right breast. SLE was confirmed based on the 2019 European League Against Rheumatism/American College of Rheumatology (EULAR/ACR) criteria. IgG-κ type multiple myeloma was confirmed by bone marrow biopsy, and the patient was synchronously diagnosed with SLE and MM after BC treatment.

**Interventions:**

Glucocorticoids and immunosuppressive agents, including intravenous hydrocortisone (5 g every 8 hours) and oral hydroxychloroquine (Plaquenil) (200 mg twice daily) were administered to treat SLE. One capsule of thalidomide 50 mg was administered orally every night at bedtime for MM.

**Outcomes:**

The patient died two days later, shortly after the administration of drugs, due to multiple organ failures secondary to pneumonia and respiratory failure.

**Conclusion:**

This is a case of MM and SLE after BC treatment. The present challenge was the early detection and accurate diagnosis of the secondary major illnesses, as the clinical manifestations were similar and non-specific between these two diseases. Awareness and prompt recognition of the common clinical symptoms of SLE and MM should be considered by clinical physicians to avoid delayed diagnoses and facilitate early treatment for a better prognosis.

## 1. Introduction

Breast cancer (BC) is the most commonly diagnosed cancer and the fifth leading cause of cancer death worldwide.^[1]^ Systemic lupus erythematosus (SLE) is characterized by the aberrant activity of the immune system, wherein the immune system starts to attack healthy cells and tissues throughout the body, leading to variable clinical symptoms.^[2]^ Multiple myeloma (MM) is an incurable, genetically heterogeneous disease of the plasma cells that is characterized by the overproduction of monoclonal immunoglobulin in the bone marrow.^[3]^ Simultaneous diagnosis of MM and SLE is rare and unusual, while the coexistence of three major diseases, such as BC, MM, and SLE, is even rarer. The symptoms reported by patients with MM and SLE on initial presentation are often nonspecific and mimic viral syndromes,^[2,4]^ resulting in considerable diagnostic challenges for clinicians outside of oncology and rheumatology. Nevertheless, to the best of our knowledge, there has been no published case report to date describing the synchronous diagnosis of MM and SLE after BC treatment. Therefore, the purpose of this case report is to describe a very rare case of SLE and MM coexistence after neoadjuvant chemotherapy combined with target therapy for BC; this will enhance clinical physicians’ awareness of early detection and the differential diagnoses of comorbid SLE and MM.

## 2. Case presentation

**Figure 1.**
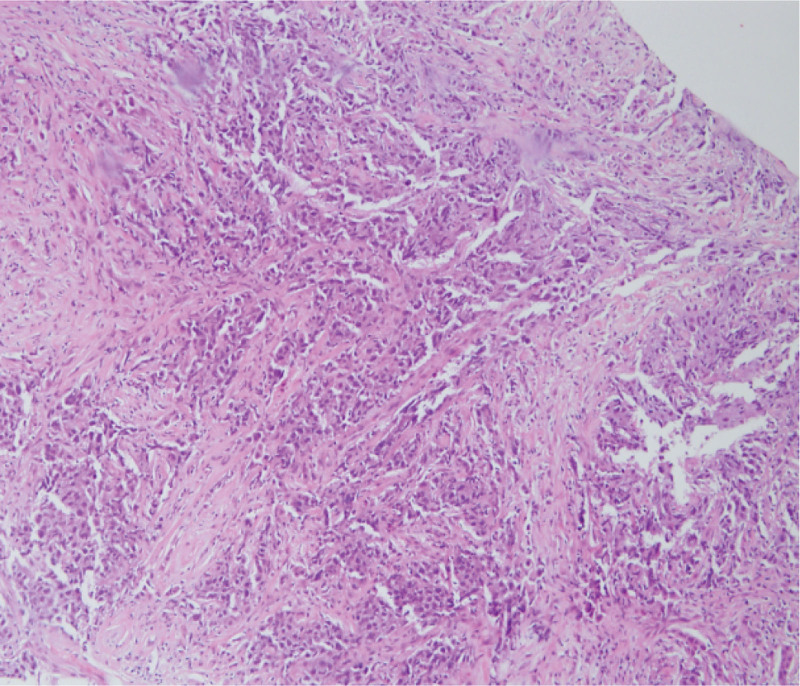
Right breast core needle biopsy shows grade 3 invasive carcinoma of no special type, characterized by diffuse infiltration of neoplastic cells without tubule formation (H&E stain, ×200).

A 69-year-old woman with a history of hypertension and hyperlipidemia was admitted to the hospital with a fever, which lasted for 2 days. She had undergone a thyroidectomy, abdominal total hysterectomy, and bilateral salpingo-oophorectomy in the distant past (surgery dates were excluded from the medical record). Approximately 8 months before admission, in November 2019, she complained of a painful mass in the right breast for 2 days. Breast sonography disclosed an irregular mass on R9/3 and L12/3 and enlarged bilateral lymph nodes. Core needle biopsy of the right breast mass confirmed invasive carcinoma of no special type with estrogen receptor (ER: 0%), progesterone receptor (PR: <1%), human epidermal growth factor receptor 2 (Her-2/Neu positive: 90%), and Ki-67 (80%) (Fig. 1). The left breast mass proved to be an ER negative (0%), PR negative (0%), lobular carcinoma in situ. Fine needle aspiration of the right axillary lymph node showed metastatic carcinoma. The clinical staging of the right breast was cT2N1aM0, cStage IIB, and the molecular subtype was HER2. Breast cancer treatment was started with neoadjuvant chemotherapy (docetaxel and carboplatin) combined with a dual HER2 blockade (trastuzumab and pertuzumab) for six courses. (The full dose of the six-course neoadjuvant therapy included docetaxel 696 mg, carboplatin AUC 36, trastuzumab 3600 mg, and pertuzumab 2940 mg).

**Figure 2.**
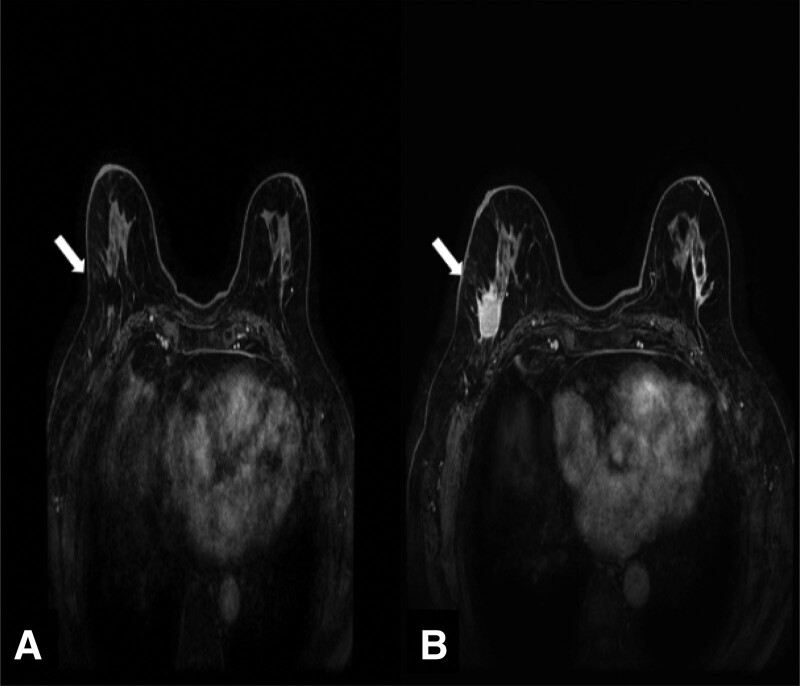
Breast magnetic resonance imaging (MRI) shows (A) an almost complete response to neoadjuvant therapy (18 mm). (B) Initial diagnosis of right breast cancer (46 mm).

After 5 months of treatment, preoperative breast magnetic resonance imaging (MRI) showed an almost complete response to neoadjuvant therapy (Fig. 2). The patient subsequently underwent bilateral total mastectomy and sentinel lymph node removal of the right axillary lymph node. The final pathology report showed residual high-grade ductal carcinoma in situ in the right breast and lobular carcinoma in situ in the left breast.

Three weeks after the bilateral mastectomy, the patient continued the adjuvant treatment of the dual HER2 blockade with trastuzumab (600 mg) and pertuzumab (420 mg) every three weeks from May 7, 2020. However, after the eighth cycle of the double HER2 blockade, she developed a localized rash and painful mass on her left buttock. Weakness and dizziness were also noted. Herpes zoster was diagnosed and lasted for 1 month; subsequently, antiviral therapy with acyclovir cream was administered for 2 weeks before this admission. Moreover, the patient suffered from fever, rhinorrhea, hoarseness, itching skin, and rashes over her trunk and limbs. She was brought to the emergency room by her family on November 14, 16, and 17, 2020. Chest X-ray (CXR) revealed left pleural effusion. Laboratory tests showed the following findings: white blood cell count, 7.36 × 10^3^/µL; band neutrophils, 19%; segmented neutrophils, 36%; platelets, 65000/µL; hemoglobin (Hb), 8.1 g/dL; sodium (Na), 130 mmol/L; potassium (K), 3.5 mmol/L; calcium (Ca), 2.07 mmol/L; creatinine, 1.1 mg/dL; and C-reactive protein (CRP), 5.33 mg/dL. We planned for her hospitalization immediately.

Two days after admission, a persistent, erythematous bilateral tibial rash was observed; however, no active arthritis was found on the hand joints, toes, elbow, and knee joints. Additional symptoms included anorexia and abdominal pain. The blood examination revealed the following results: blood urea nitrogen (BUN), 18 mg/dL; creatinine, 1.5 mg/dL; platelets, 21 × 10^3^/µL; erythrocyte sedimentation rate (ESR), 43 mm/h; Hb, 8.1 g/dL; and Ca, 2.07 mmol/L. Urinalysis showed proteinuria (protein: 300 mg/dL, 3+, granular cast: 6–9) and hematuria (occult blood [OB] 1+). The 24-h urine protein was 4.4 g. CXR revealed bilateral syndrome and septic shock. Antibiotic treatment with imipenem 250 mg every 6 h and teicoplanin 400 mg every 12 h were used for infection control, along with a diuretic agent, antihistamines, blood transfusion, resuscitation, and a vasopressor agent. However, acute renal failure, followed by azotemia and refractory thrombocytopenia, was observed. The first hemodialysis session was performed on November 23, 2020, and then every 2 days thereafter. Physical examination showed multiple subcutaneous skin rashes over the patient’s trunk and limbs. An allergy test investigating the presence of immunoglobulins, such as immunoglobulin A (IgA), immunoglobulin G (IgG), immunoglobulin M (IgM), and immunoglobulin E (IgE), revealed a high IgG level (>3000 mg/dL). The hematologist and rheumatologist were consulted to determine the differential diagnosis of suspected autoimmune disease or MM. Extended blood tests and a series of immunological studies were obtained. The level of anti-nuclear antibodies was more than 1280 times greater than the normal value. Anti-cardiolipin-IgM, anti-β2GP1, and lupus anticoagulant were all positive. C3 and C4 levels were 81.5 and <8 mg/dL, respectively (Table 1). Lupus was confirmed according to the 2019 European League Against Rheumatism/American College of Rheumatology (EULAR/ACR) criteria.

**Table 1:** Laboratory findings aiding the diagnosis of systemic lupus erythematosus.

Blood Test	Data
ANA	>1:1280
Anti-dsDNA	Negative
Anti-cardiolipin-IgM	Positive
Anti- β2GP1 antibodies	Positive
LACscreen (s)	68.7
C3	81.5
C4	<8
Urine	Data
OB (mg/dL)	0.06 (1+)
PRO (mg/dL)	300 (3+)
Granular cast (/LPF)	6-9
Creatinine (mg/dL)	35.13
Total protein (24 h urine) (mg/d)	4406.22

ANA = antinuclear antibody, Anti dsDNA = antinuclear double-strand DNA, Anti- β2GP1 antibodies = anti-beta 2 glycoprotein-1 antibodies, C3 = Complement 3, C4 = Complement 4, LAC = Lupus anticoagulant, OB = occult blood, PRO = protein.

**Figure 3.**
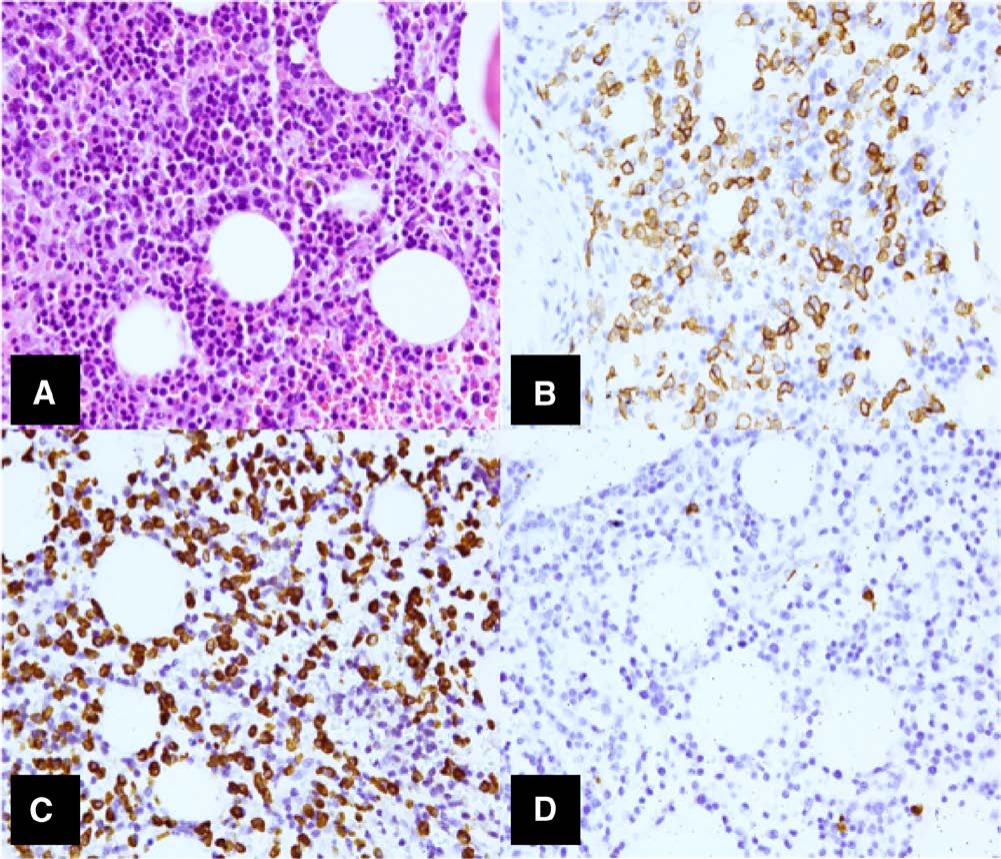
Immunohistochemical staining of myeloma cells obtained from the bone marrow biopsy. (A) H&E stain, ×400; (B) CD138 by immunohistochemistry, ×400: >10% of plasma cells are observed by bone marrow biopsy; (C) Kappa light chain-ISH, ×400 (D) Lambda light chain-ISH, ×400: Plasma cells show kappa light chain restriction.

**Figure 4.**
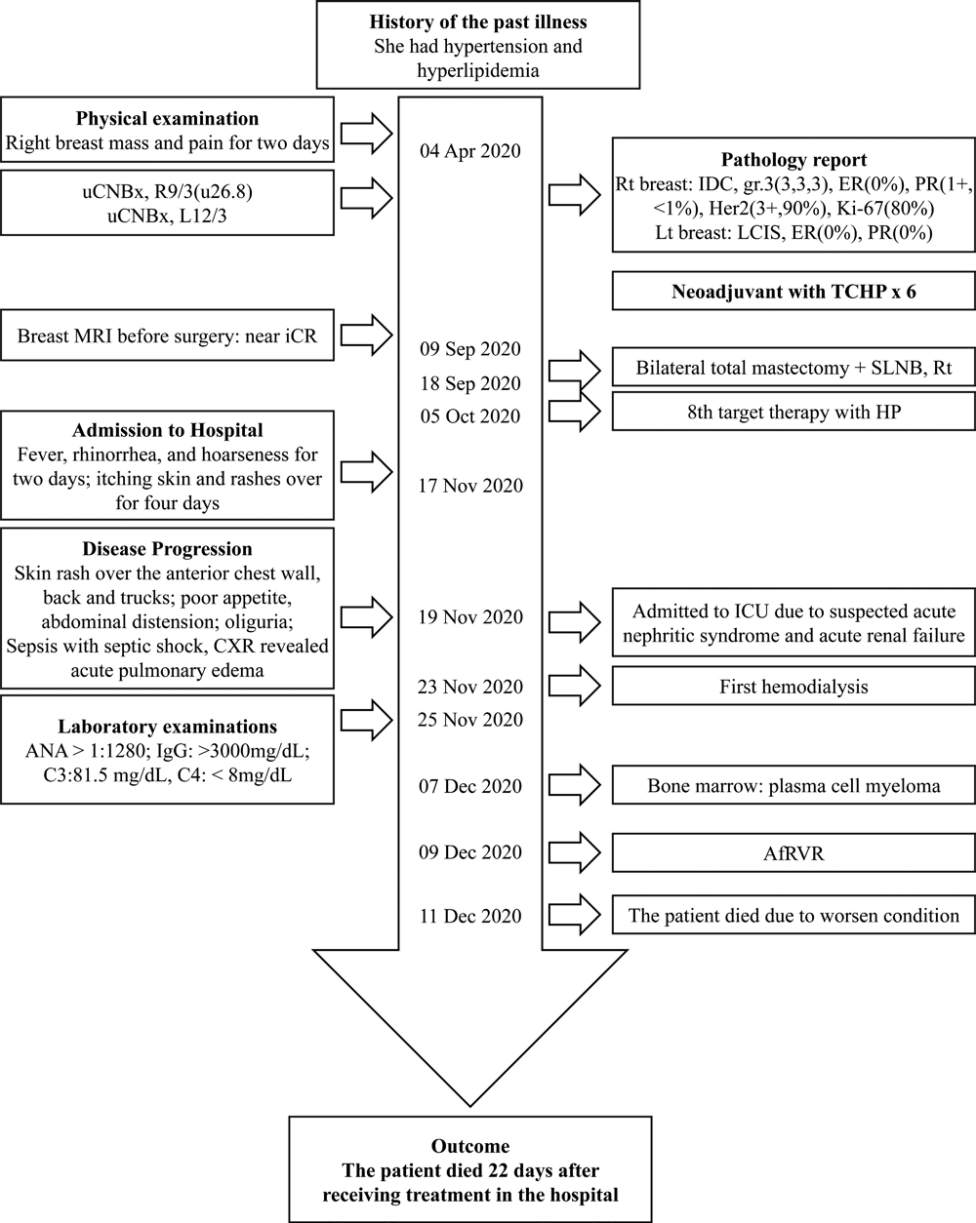
Case report timeline presented according to CARE guidelines.

Conversely, the serum immunofixation electrophoresis (IFE) test showed an increased IgG >3000 mg/dL and normal IgA and IgM levels of 114 mg/dL and 254.5 mg/dL, respectively. The test found an r-globulin level of 5140 mg/dL, β-globulin level of 1040 mg/dL, and M-protein level of 33.56%. The free kappa light chain (FKLC) and free lambda light chain (FLLC) were 53437.5 mg/L and 345 mg/L, respectively, with a K/L ratio of 157.89. The level of the serum IgE was 335/mL and that of lactate dehydrogenase (LDH) was 424 IU/L (Table 2). Bone marrow aspiration showed at least 8% monoclonal plasma cells with cytoplasmic kappa light chain restriction. To confirm the diagnosis, a bone marrow biopsy was performed and revealed that plasma cells exceeded 10% of the marrow nucleated cells. The ancillary studies of the plasma cell showed monoclonality for the kappa light chain (Fig. 3). Based on the examination results, the disease was classified as stage 3 according to the Revised International Staging System for Myeloma.^[5]^ The final diagnosis was IgG-κ type MM, SLE, and ductal carcinoma in situ of the right breast. A diagnosis of SLE was established on December 3, 2019, and MM was diagnosed on December 10, 2019. Administration of intravenous hydrocortisone (50 mg every 8 hours) and hydroxychloroquine (Plaquenil) (200 mg twice a day per oral) was started on December 4, 2020, for SLE treatment. One capsule of thalidomide was orally administered every night at bedtime for MM treatment from December 10, 2020. Unfortunately, the patient died two days after the confirmed diagnosis of MM because of multiple organ failure caused by pneumonia and respiratory failure. This timeline is shown in Figure 4.

**Table 2:** Laboratory findings aiding the diagnosis of multiple myeloma.

Blood Test	Data
Hgb (g/dL)	8.4
Hct(%)	24.7
Platelet (*10^3^/µL)	21
Creatinine (mg/dL)	1.5
Ca (mmol/L)	2.07
Albumin (g/dL)	2.8
LDH (U/L)	424
ESR (mm/h)	43
CRP (mg/dL)	5.33
FKLC (mg/dL)	53437.5
FLLC (mg/dL)	345
kappa/lambda ratio	154.89
IgG (mg/dL)	>3000

Ca = calcium, CRP = C-reactive protein, ESR = erythrocyte sedimentation rate, FKLC = free kappa light chain, FLLC = free lambda light chain, Hct = hematocrit, Hgb = hemoglobin, IgG = immunoglobulin G, LDH = lactate dehydrogenase.

## 3. Discussion

According to the newest global cancer statistics report, there are 2,261,419 female BC cases, which account for 11.7% of all cancer cases; this has surpassed lung cancer as the most commonly diagnosed cancer.^[1]^ SLE is most prevalent in women of childbearing age, between the ages of 15 and 44 years old.^[6,7]^ It is a multiorgan disease that can mimic infectious diseases, cancer, and other autoimmune conditions that need a combination of typical clinical presentation and positive serologies for diagnosis.^[2]^ The clinical manifestation of SLE is heterogenous and may range from fatigue and joint pain to severe and life-threatening organ damage. Weight loss, low-grade fever, fatigue, arthralgias, and arthritis are also common constitutional symptoms.^[2]^ Additionally, cutaneous manifestations occur in 75%–80% of SLE patients.^[8]^ Moreover, 30%–60% of all SLE patients initially present with renal disease^[9]^; primary or secondary involvement of the respiratory system may also occur in 50%–70% of SLE patients.^[10]^ Because of the variety of clinical presentations, the international SLE-EULAR/ACR criteria are commonly used for SLE diagnosis.^[2]^ MM is the third most common hematologic malignancy, accounting for 0.9% of all cancer cases.^[1]^ The median diagnosis age of MM is 65 years, with a 5-year survival rate of approximately 46.6%–55.6%.^[3,11,12]^ The typical clinical presentations are known as the CRAB criteria and include hypercalcemia, renal insufficiency, anemia, and bone lesions.^[3]^ However, just like SLE, beyond the usual CRAB symptoms, a significant proportion of patients with MM present with a variety of other clinical manifestations, which may increase the diagnostic difficulty,^[4]^ especially when patients experience multiple medical problems. Furthermore, approximately 50.6% of MM patients visited their general practitioner three or more times before a hospital referral compared with patients with other cancer types.^[13]^

In this case report, the patient presented with three distinct diseases as follows: BC, SLE, and MM. Although all three diseases were diagnosed at the age of 69 years, SLE and MM were diagnosed 8 months after the nearly complete tumor response for BC treatment. In this case, the diagnosis of SLE was based on 2019 EULAR/ACR criteria,^[2]^ which included the following: (1) leukopenia; (2) thrombocytopenia; (3) pleural effusion; (4) proteinuria; (5) high-titer ANA; (6) positive anti-cardiolipin-IgM, anti-β2 GP1 antibodies, and lupus anticoagulant; and (7) low C3 and C4 levels. The diagnosis of the IgG-κ type MM was based on (1) elevated serologic IgG (> 3000 mg/dL), (2) paraprotein band of the r-globulin level of 5140 mg/dL, β-globulin of 1040 mg/dL and M-protein of 3020 mg/dL by IFE, (3) increased numbers of plasma cells (20%–25%) in bone marrow biopsy, and (4) a kappa/lambda ratio of 154.89.

Compared with the previous literature, the diagnosis age of this case is quite different for SLE but compatible with MM.^[2,3]^ The clinical manifestations upon initial presentation of this patient were fever, abdominal pain, and anorexia; these are common and non-specific symptoms that may interfere with the diagnosis of the two diseases. Moreover, SLE and MM often share the same clinical manifestations, such as anemia, leukopenia, thrombocytopenia, and renal involvement. According to the literature, the typical clinical manifestations of MM majorly include anemia, bone lesion, infections, hypercalcemia, renal failure, fatigue, and pain,^[3]^ all of which were observed in our case, except for bone lesion and hypercalcemia. Another cutaneous manifestation may occur in 75%–80% of SLE patients;^[2]^ however, this was not observed in this case. No indurated or flat erythematous lesions were found on the malar eminences, scalp, arms, hands, neck, or chest of this patient; only erythematous rashes over the bilateral tibial area were observed. The aforementioned symptoms may increase the difficulty of early diagnosis of the two diseases. Nevertheless, evidence shows a concurrent diagnosis of MM and SLE is a rare and uncommon event; upon performing a literature review through PubMed, only three case reports were found,^[14–16]^ although there were some reports of an increased risk of hematological malignancies after diagnosis or treatment for BC survivors.^[17,18]^ A retrospective nested case-control study based on the National Health Insurance Research Database (NHIRD) conducted in Taiwan included 14842 SLE patients and analyzed the risk of malignancy among SLE patients. The study found that the risk of malignancy was associated with cumulative exposure to immunosuppressants. In total, 330 patients developed malignancy, with BC among the most frequently diagnosed cancers, accounting for 16.9% of the included cases.^[19]^ However, in this case, SLE was diagnosed after BC treatment, and the patient was not administered immunosuppressants. Jabagi et al^[17]^ reported that the increased risk of hematologic neoplasms, including MM, is related to BC treatment modalities such as chemotherapy and radiotherapy. The same claim was found in a study by Gürel et al^[20]^ However, in our case, the patient did not receive radiotherapy; rather, she received docetaxel, carboplatin combined with trastuzumab, and pertuzumab. In the literature review, we only found one report by Marinopoulos et al. that described an MM case emerging six months after a chemotherapy regimen of cisplatin, docetaxel, vinorelbine, and topotecan for non-small-cell lung cancer.^[21]^ No case reports on carboplatin-, trastuzumab-, and pertuzumab-related secondary hematologic neoplasms were found. To the best of our knowledge, there has been no case report published to date describing the synchronous diagnosis of MM and SLE after BC treatment. This case illustrated the similar manifestations between MM and SLE upon initial presentation and highlighted the speed of disease progression leading to multiple organ failure and mortality.

Although the etiology and the possible pathogenetic mechanisms underlying the association among these three diseases remain unclear, we should recognize that the development of more than one aggressive major disease in the patient after BC treatment, unspecific symptoms such as anemia, unknown cause of infection, and acute renal failure should be considered for differential diagnosis of MM and SLE to avoid delay in diagnosis and early treatment.

## Acknowledgments

We would like to thank Tai Yu Jen, a nurse practitioner, for his assistance with data collection, as well as Dr. Chao-Yuan Yao for comments that greatly improved the manuscript.

## References

[1] Sung H Ferlay J Siegel RL. Global Cancer Statistics 2020: GLOBOCAN estimates of incidence and mortality worldwide for 36 cancers in 185 countries. CA Cancer J Clin. 2021;71:209–49. 33538338 10.3322/caac.21660

[2] Kiriakidou M Ching CL. Systemic lupus erythematosus. Ann Intern Med. 2020;172:ITC81–ITC96. 32479157 10.7326/AITC202006020

[3] Brigle K Rogers B. Pathobiology and diagnosis of multiple myeloma. Semin Oncol Nurs. 2017;33:225–36. 28688533 10.1016/j.soncn.2017.05.012

[4] Talamo G Farooq U Zangari M. Beyond the CRAB symptoms: a study of presenting clinical manifestations of multiple myeloma. Clin Lymphoma Myeloma Leuk. 2010;10:464–8. 21156463 10.3816/CLML.2010.n.080

[5] Rajkumar SV. Updated diagnostic criteria and staging system for multiple myeloma. Am Soc Clin Oncol Educ Book. 2016;35:e418–23. 27249749 10.1200/EDBK_159009

[6] Chen SY Hsu CH. To investigate the age at onset and gender distribution of patients with systemic lupus erythematosus in Taiwan. J Health Manag. 2020;18:79–90.

[7] Fava A Petri M. Systemic lupus erythematosus: diagnosis and clinical management. J Autoimmun. 2019;96:1–13. 30448290 10.1016/j.jaut.2018.11.001 PMC6310637

[8] Vera-Recabarren MA García-Carrasco M Ramos-Casals M. Comparative analysis of subacute cutaneous lupus erythematosus and chronic cutaneous lupus erythematosus: clinical and immunological study of 270 patients. Br J Dermatol. 2010;162:91–101. 19785596 10.1111/j.1365-2133.2009.09472.x

[9] Aragón CC Tafúr RA Suárez-Avellaneda A. Urinary biomarkers in lupus nephritis. J Transl Autoimmun. 2020;3:100042. 32743523 10.1016/j.jtauto.2020.100042 PMC7388339

[10] Hannah JR D’Cruz DP. Pulmonary complications of systemic lupus erythematosus. Semin Respir Crit Care Med. 2019;40:227–34. 31137062 10.1055/s-0039-1685537

[11] Firth J, Medical Masterclass contributors. Haematology: multiple myeloma. Clin Med (Lond). 2019;19:58–60. 30651246 10.7861/clinmedicine.19-1-58 PMC6399642

[12] Surveillance, Epidemiology, and End Results (SEER) Program. SEER Cancer Stat Facts: Myeloma. Available at: https://seer.cancer.gov/statfacts/html/mulmy.html. [Access date September 6, 2021].

[13] Lyratzopoulos G Neal RD Barbiere JM. Variation in number of general practitioner consultations before hospital referral for cancer: findings from the 2010 National Cancer Patient Experience Survey in England. Lancet Oncol. 2012;13:353–65. 22365494 10.1016/S1470-2045(12)70041-4

[14] Pehamberger H Diem E Konrad K. Systemic lupus erythematosus with multiple myeloma. Acta Derm Venereol. 1978;58:527–30. 83077

[15] Vaiopoulos G Konstantopoulos K Mantzourani M. Multiple myeloma associated with systemic lupus erythematosus. Leuk Lymphoma. 2003;44:373–4. 12688362 10.1080/1042819021000037949

[16] Humayun M Haider I Khan A. SLE and multiple myeloma, a rare and unusual association. J Med Sci. 2014;22:96–8.

[17] Jabagi MJ Goncalves A Vey N. Risk of hematologic malignant neoplasms after postoperative treatment of breast cancer. Cancers (Basel). 2019;11:1463. 10.3390/cancers11101463 PMC6827362 31569513

[18] Jabagi MJ Vey N Goncalves A. Evaluation of the incidence of hematologic malignant neoplasms among breast cancer survivors in France. JAMA Netw Open. 2019;2:e187147. 30657534 10.1001/jamanetworkopen.2018.7147 PMC6484549

[19] Hsu CY Lin MS Su YJ. Cumulative immunosuppressant exposure is associated with diversified cancer risk among 14 832 patients with systemic lupus erythematosus: a nested case-control study. Rheumatology (Oxford). 2017;56:620–8. 28039419 10.1093/rheumatology/kew457

[20] Gürel A Aygen B Kara M. Multiple myeloma emerging after chemotherapy for breast cancer: case presentation and a brief review. Van Tip Derg Med J. 2015;23:194–6.

[21] Marinopoulos S Skorda L Karatapanis S. Multiple myeloma emerging after chemotherapy for non-small-cell lung cancer. Med Oncol. 2008;25:415–8. 18345519 10.1007/s12032-008-9056-0

